# Nectar discovery speeds and multimodal displays: assessing nectar search times in bees with radiating and non-radiating guides

**DOI:** 10.1007/s10682-017-9916-1

**Published:** 2017-08-10

**Authors:** David A. Lawson, Heather M. Whitney, Sean A. Rands

**Affiliations:** School of Biological Sciences, University of Bristol, Bristol, BS8 1TQ UK

**Keywords:** Floral displays, Pollination, Spatial fragrance patterns, Plant-pollinator coevolution

## Abstract

Floral displays are often composed of areas of contrasting stimuli which flower visitors use as guides, increasing both foraging efficiency and the likelihood of pollen transfer. Many aspects of how these displays benefit foraging efficiency are still unexplored, particularly those surrounding multimodal signals and the spatial arrangement of the display components. We compare the nectar discovery times of forager bumblebees (*Bombus terrestris*) when presented with artificial flowers with unimodal or compound displays of visual and/or olfactory stimuli, positioned in either radiating or non-radiating arrangements. We found that the addition of individual display components from either modality reduces nectar discovery time but there was no time benefit to bimodal displays over unimodal displays or any benefit to radiating stimuli arrangements over non-radiating arrangements. However, preference tests revealed a time advantage to radiating unimodal visual patterns over non-radiating unimodal visual patterns when both types were displayed simultaneously. These results suggest that the benefits of multimodal stimuli arrangements to pollinators are unrelated to benefits in nectar discovery time. Our results also suggest that spatial patterns of scent can be used as nectar guides and can reduce nectar discovery times without the aid of visual stimuli.

**Electronic supplementary material:**

The online version of this article (doi:10.1007/s10682-017-9916-1) contains supplementary material, which is available to authorized users.

## Introduction

The initial means of communication between flowering plants and their visitors are visual and olfactory signals (Cook et al. 2002; Raguso 2004; Balao et al. 2011). A well-studied and taxonomically widespread feature of these displays are nectar guides which were first described by Sprengel (1793). Nectar guides are contrasting patterns of floral stimuli thought to minimise nectar discovery times, increase the foraging efficiency of flower visitors and increase the rate at which pollinators transfer pollen between conspecifics (Penny 1983; Waser and Price 1983; Leonard and Papaj 2011; Hansen et al. 2012). Nectar guides have been seen to increase the relative frequency of legitimate flower visits in flowers at risk of nectar robbing (Leonard et al. 2013) and may also broaden the range of pollinator species that visit a flower (Ollerton et al. 2007).

**Fig. 1:**
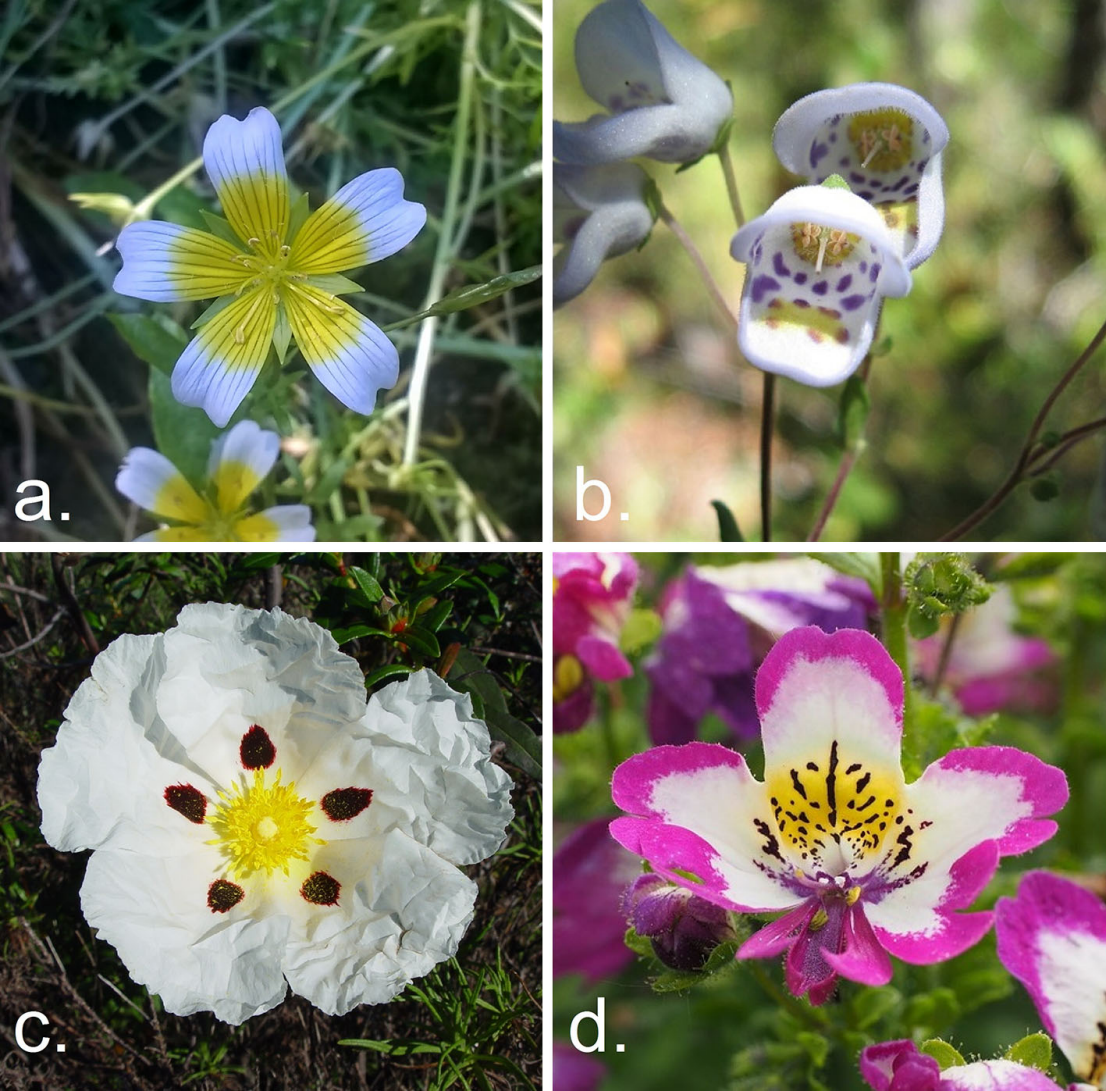
Examples of different types of visual nectar guides. **a** *Limnathes douglasii,* demonstrating *straight*, *thin* nectar guides and centre with a *contrasting colour*; **b** *Jovellana punctate*, demonstrating a non-radiating speckled guide; **c** *Cistus ladanifer*, demonstrating peripheral *dots* of *contrasting colour*; **d** *Schizanthus wisetonensis,* demonstrating speckled flowers with a radiating arrangement. Photo credits: a. David Lawson; b. Pabloendemico; c. Carsten Niehaus; d. Hans Braxmeier. See image references for full details

The most widespread of these cues contain markings around the corolla openings, peripheral dots or lines which radiate from the nectary (Fig. 1) (Proctor et al. 1996; Dafni et al. 1997), with the use of these guides being observed in bees, hummingbirds, hawkmoths and syrphid flies (Dinkel and Lunau 2001; Knoll 1926; Waser and Price 1983). These radiating lines, whereby the pattern spreads linearly outwards from the floral reward (Fig. 1a), have been shown to increase the attractiveness of flowers over others which do not have them (Manning 1956; Free 1970; Dinkel and Lunau 2001; Leonard and Papaj 2011; Leonard et al. 2013). Considering the potential benefits to nectar discovery times and accuracy, these preferences are unsurprising given that even small increases in the rate at which nectar is collected can scale up to increase the reproductive success of the entire colony (Pelletier and McNeil 2003). Bees have also been shown to visit artificial flowers with visual nectar guides when the flowers no longer offered a reward, but did not do so for plain flowers, highlighting the benefit of nectar guides to plants which can receive visits regardless of reward status (Leonard and Papaj 2011). Removal of nectar guides in iris *Lapeirousia oreogena* also reduced pollen analogue export and fruit set, suggesting nectar guides are under strong selective maintenance through effects to both male and female fitness (Hansen et al. 2012).

Although previous studies have compared search times between radiating guides and plain flowers (Leonard et al. 2011a, b; Waser and Price 1983), it is unknown if radiating guides would reduce nectar discovery times over non-radiating patterns (Fig. 1b). This radiating aspect of nectar guides appears to be important in terms of flower orientation to species such as bombyliid flies and bumblebees (Johnson and Dafni 1998; Goodale et al. 2014). Considering these points, it would be of interest to explore the differences between these pattern types.

These visual guides are not the only orientation cues used by flowering plants. Many species produce scented nectar, as investigated by Raguso (2004). Distinct pollen odours have also been observed in multiple species (Dobson et al. 1990, 1996; Bergström et al. 1995). These scented rewards provide an honest signal to flower visitors and may serve as an olfactory flag providing another means by which flower visitors can orientate within the flower (Bergström et al. 1995). Flowers can also have an uneven distribution of scent within and between floral organs. For example, the nectar-producing petal areas in *Ranunculus acris* produced more volatiles than the areas which did not produce nectar (Bergström et al. 1995). These spatial fragrance patterns are believed to act as orientation cues on flowers, acting as olfactory nectar guides and have been observed in a wide range of species (Bergström et al. 1995; Raguso and Pichersky 1999; Flamini et al. 2002). However, reductions in nectar discovery time through the use of spatial fragrance patterns has not been shown under experimental conditions. These visual guides and spatial fragrance patterns may occur in spatially corresponding locations on the flower, and studies have found that different colour morphs of certain species have different scent profiles (Flamini et al. 2002; Salzmann and Schiestl 2007; Zuker et al. 2002). However, this relationship between colour morphs and scent profile is not observed in all species (Olesen and Knudsen 1994; Dormont et al. 2010), making it difficult to say how ubiquitous these relationships are.

It is currently understood that multimodal displays have considerable effects on the learning and efficiency of foraging pollinators (Hebets and Papaj 2005; Goyret et al. 2007; Kulahci et al. 2008; Katzenberger et al. 2013). Additionally, visual nectar guides are known to reduce the time foragers spend on flowers (Leonard and Papaj 2011). However, it is currently unknown whether nectar guides which incorporate display components from multiple modalities are more effective than unimodal guides (i.e. guides which are comprised of only one sensory modality). Hebets and Papaj (2005) suggested that the function of multimodal signals may relate to inter-signal interactions where the presence of one signal acts to increase the probability and/or speed of detection of a second signal (the ‘increased detection and discrimination hypothesis’). If this is the case, foragers presented with bimodal nectar guides (i.e. guides which are comprised of components from two sensory modalities) may have increased detection of one aspect of the display leading to a potentially shorter nectar discovery time when compared to unimodal guides. Aside from time benefits to the pollinator, easier access to nectar though decreased nectar discovery times may increase the range of possible pollinators for a plant (Ollerton et al. 2007), minimise loss of pollen or potentially regulate the amount of pollen deposited on visitors (Harder and Thomson 1989; Leonard and Papaj 2011). Bimodal nectar guides present us with an opportunity to investigate foraging speeds in a multimodal context whilst looking into the potential interactions these guides have with other aspects of floral displays, such as the radiating or non-radiating arrangement of these patterns.

Within this study, we examined the speed of *Bombus terrestris* foragers while locating feeding wells on artificial flowers when presented with either radiating patterns or non-radiating patterns comprised of visual stimuli, olfactory stimuli or both. We investigated the preferences individual foragers had for these pattern combinations. We hypothesised that the time it takes a forager bee to locate feeding wells would be shorter in radiating patterns compared to non-radiating patterns and shorter in bimodal displays over unimodal displays. These hypotheses were tested in speed comparison experiments in which the time between landing and feeding from the nectary of forager *B. terrestris* was recorded. Pre-landing preferences were recorded in separate experiments. Within this study, we define nectar discovery time (hereafter NDT) as the time between landing and proboscis extension into the central feeding well.

## Method

### Flight arena, bumblebee colony conditions and animal welfare

All experiment types were carried out in wooden framed 72 × 104 × 30 cm flight arenas topped with UV-transparent perspex with the floor covered in Advance Green gaffer tape (Stage Electrics, Bristol, UK). Flight arenas were connected to the plastic nesting box of flower naïve *Bombus terrestris* subsp. *audax* (Harris, 1776) colonies (Koppert BV, Berkel en Rodenrijs, Netherlands and Syngenta-Bioline, Little Clacton, UK) via a transparent gated tube which could be manually manipulated to regulate which bees, and how many, could enter or leave the flight arena. Six Sylvania Activa 172 Professional 36 W fluorescent tubes (Havells-Sylvania Germany GmbH, Erlangen, Germany) on a 12-hour light/dark regime were used to simulate natural illumination. Bees were fed 30% sucrose solution daily ad libitum after experiments had taken place and pollen was added directly to the colony three days a week. Foraging individuals were marked on their thorax with an identifying pattern of non-toxic paint before experiments took place. All work conforms to the legal requirements of the UK where it was carried out and conforms to the welfare requirements of both the UK and ASAB/ABS Guidelines for the Use of Animals in Research.

### Artificial flowers

**Fig. 2:**
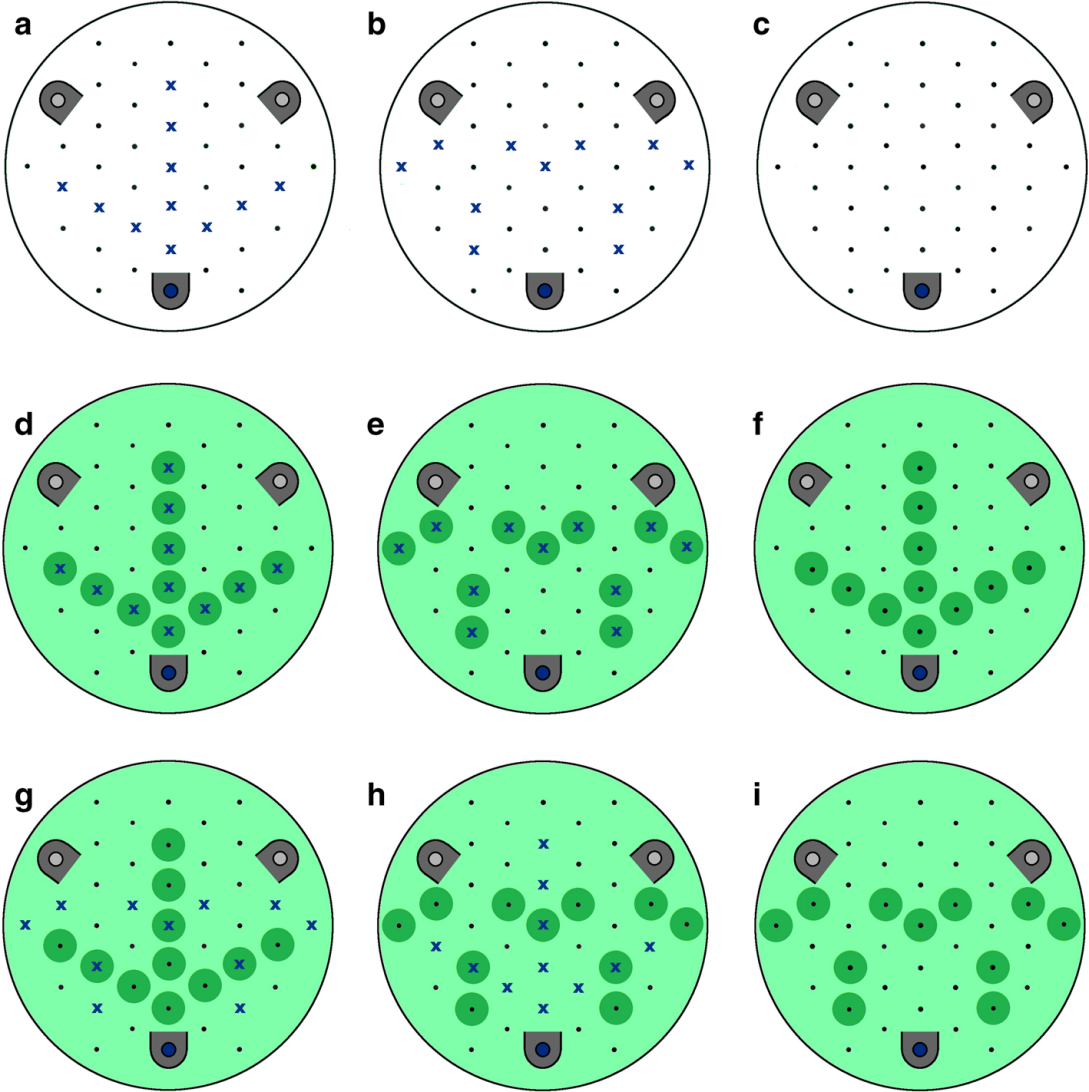
Artificial flowers used during the no-choice speed test. Visual patterns are shown and scent placements are shown with x’s. **a** Unimodal scented—radiating; **b** Unimodal scented—non-radiating; **c** Scentless and colourless control; **d** Bimodal—radiating; **e** Bimodal—non-radiating; **f** Unimodal visual—radiating; **g** Bimodal—radiating visual, non-radiating scent; **h** Bimodal—non-radiating visual—radiating scent and; **i** Unimodal visual—non-radiating. Placement of the 3 lid wells and cover structure is also displayed with the dark feeder at the *bottom* of each disc being the location which contains sucrose. More *contrasting colours* were used here to more easily view pattern arrangements

6 white perspex discs (80 mm diameter, 3 mm width) were used as foraging stimuli during each experiment. Each disc had 43 holes (2 mm diameter) in a hexagonal pattern (Fig. 2). Plastic covers with holes corresponding to those of the discs were placed on the top of each disc. Each of these covers had either a printed visual pattern or no visual pattern (Fig. 2). The back of each cover had an additional layer of self-adhesive film in order to prevent spoilage on the printed pattern and to allow for the easy removal of volatiles after experiments. At the start of each experimental session a layer of self-adhesive covering film was placed on the bottom of each Perspex disc so the holes can contain small amounts of liquid. At the end of each day this film was removed and the discs were soaked overnight in a detergent solution to remove volatiles and glue residue.

Each film had 43 holes (2 mm diameter) corresponding to those on the disc and three upturned lids of 0.5 ml Eppendorf containers glued to three equidistant points on the disc surface to be used to contain sucrose or to be left empty. Each of these upturned lids was painted (white for blank discs, green for visual pattern discs) to reduce visibility of contents and each had a plastic hood placed on top which meant bees could only access the feeding wells from one direction (shown in Fig. 2). All visual patterns consisted of a green background of hue 140° HSB (140;50;100) with a different arrangement of green circles of 120° HSB (140;50;100) (10 mm diameter) around 11 of the holes. These two similar greens, which have been previously used in Leonard et al. (2011a, b), Clarke et al. (2013) and similar unpublished experiments (Lawson in prep.) with encouraging results, were chosen so that the pattern was not immediately obvious to foragers from a distance. More obvious guides could cause bees to land close to the nectary and limit their exposure to the flower’s scent patterns (Lunau et al. 2006).

Alongside the visual patterns presented, there were also sets of discs loaded with scent patterns corresponding to the arrangement of the visual patterns. In these discs, a pattern of peppermint oil solution was also presented (a 1:10 mix of peppermint oil: mineral oil, where the peppermint essential oil came from Amphora Aromatics, Bristol, UK), whereby 2.5 µL of the peppermint solution was pipetted into eleven of the wells in a radiating or non-radiating pattern which spatially matched the visual patterns (Fig. 2). Given the three types of visual pattern (radiating, non-radiating and a control) and three corresponding scent patterns, there were nine possible combinations of visual and scent pattern combined. All nine of these pairings were considered in the experiment, with fourteen foragers used in each pairing (126 total).

### No-choice speed test

In each experiment, the flight arena was cleared of bees and cleaned. Six discs of the same type were placed in the flight arena with a 30% sucrose solution (20 μL) placed in the appropriate well for each disc (Fig. 2). All discs were placed on top of upturned transparent plastic containers (6 cm height, 150 ml, Sterilin UK) and distributed randomly throughout the flight arena. Individual marked foragers which were naïve to both scent and visual stimuli, but had experience drinking from Eppendorf lid wells, were then allowed entry into the flight arena. The time between landing and drinking from the sucrose-containing well was measured and recorded. Once a forager had left a flower from which it had drunk, the disc was removed and sucrose was re-administered into the appropriate well and then placed back into the flight arena. The number of times a forager leaves a flower without drinking was also recorded. Once a forager had finished a foraging bout all discs were removed and swabbed with ethanol including the plastic well covers to remove visual cues and foraging pheromones and then placed into a new random distribution. This continued until the forager had landed and drunk from 30 discs. This experiment was conducted on 126 forager bees (fourteen for each disc type) from 11 colonies.

### Radiating/non-radiating preference tests

Ten discs were placed into the flight arena from one of two treatments. One treatment with five unimodal radiating visual pattern flowers and five unimodal non-radiating visual patterns and another treatment with five bimodal radiating pattern flowers and five bimodal non-radiating pattern flowers. These discs were randomly distributed with sucrose placed in the appropriate well for each disc (Fig. 2). Individually marked foragers, which were naïve to the scent and visual stimuli, were then allowed into the flight arena where their landing choice was recorded as well as the time between landing and drinking and whether a forager left a flower without drinking. This continued until the forager had landed and drunk from twenty discs. These preference tests were conducted on 60 forager bees from three colonies.

## Analysis

### No-choice nectar discovery speed test

A multiple linear regression model was used to analyse the effect of the different visual and olfactory patterns on nectar discovery time (NDT). Radiating scent, non-radiating scent, radiating visual, non-radiating visual and visit number and the interactions between these variables were used as predictors within the model, giving the linear model a structure similar to a 2-factor ANOVA. Both subject and colony were incorporated into the model as random effects. Nectar discovery times were log transformed to meet assumptions of the analysis. Welch’s two-sample *t* tests were used to compare the NDTs between foragers presented with bimodal *versus* unimodal flowers as well as between foragers exposed to radiating *versus* non-radiating floral displays in order to look at the benefits of these stimuli arrangements and combinations. Controls which had no display components were not included in these tests nor were the ‘radiating visual—non-radiating scent’ and ‘non-radiating visual—radiating scent’ groups included in the comparison test between radiating and non-radiating displays as these groups contain both aspects simultaneously. We also compared the number of times forager bees abandoned flowers before drinking between scented and unscented flowers and visual and non-visual flowers using independent 2-group *t* tests.

### Radiating and non-radiating preference tests

The preference for either radiating or non-radiating patterns in both visual and visual plus scent preference tests, measured in flower visits out of 20 (as 20 choices were made in total per forager), was compared using paired sample *t* tests after log transformations. Paired *t* tests (after log transformations) were used to compare the NDTs and the number of flower landings which did not lead to drinks between radiating and non-radiating patterns in both visual and visual plus scent preference tests.

R version 3.0.1 (R Development Core Team 2013) was used for all analysis.

## Results

### No-choice nectar discovery speed test

The multiple linear regression analysis found bumblebee nectar discovery time (NDT) was affected by all individual display components, both radiating and non-radiating and visual and olfactory, all of which reduced NDT (Table 1). There were also significant interactions between these pattern types. Flower visit number also affected the NDT, however, there was no interaction between flower visit number and the individual display components (Table 1). The interactions increased NDTs with the interaction between non-radiating scent and radiating visual patterns increasing NDT by 0.65 s from the intercept (Table 1). These interactions show that the visual components of the floral display are slowing the bees’ responses to the olfactory cues or vice versa.

**Table 1:** Effects of radiating scent, non-radiating scent, radiating visual and non-radiating visual patterns and visit number on the nectar discovery time of forager bumblebees after log transformation (multiple linear regression: *SE*_*resid*_ = 0.68, *r*_*adj*_^2^ = 0.09, *F*_13,3766_ = 29.74, *p* < 0.001)

Explanatory factor	Estimate	SE	*T*_3766_	*p*
(Intercept)	1.278	0.055	23.05	<0.001***
Radiating scent	−0.288	0.068	−4.26	<0.001***
Non-radiating scent	−0.338	0.068	−4.99	<0.001***
Radiating visual	−0.411	0.068	−6.07	<0.001***
Non-radiating visual	−0.393	0.068	−5.81	<0.001***
Visit number	−0.014	0.003	−5.00	<0.001***
Radiating scent: radiating visual	0.263	0.067	3.96	<0.001***
Non-radiating scent: radiating visual	0.647	0.067	9.72	<0.001***
Radiating scent: non-radiating visual	0.153	0.067	2.30	0.021*
Non-radiating scent: non-radiating visual	0.289	0.067	4.35	<0.001***
Radiating scent: visit number	−0.006	0.003	−1.84	0.065
Non-radiating scent: visit number	−0.005	0.003	−1.58	0.115
Radiating visual: visit number	0.001	0.003	0.17	0.867
Non-radiating visual: visit number	0.004	0.003	1.35	0.176

There was no difference in NDT between the foragers exposed to unimodal floral displays and those exposed to bimodal floral displays (unimodal: NDT = 2.73 ± 0.76 s (mean ± SD), bimodal: NDT = 2.53 ± 0.72 s, two-sample *t* test: *t*_110_ = 0.83, *p* = 0.407). There was no difference in NDT between the foragers exposed to flowers with purely radiating display elements and those exposed to flowers with purely non-radiating display elements (radiating: NDT = 2.46 ± 0.65 s, non-radiating: NDT = 2.53 ± 0.71 s, two-sample *t* test: *t*_41_ = −0.69, *p* = 0.548). The mean number of flower abandonment was larger in unscented flowers than scented flowers (unscented: 6.7 ± 4.19 flower abandonments, *N* = 30, scented: 4.79 ± 2.82 flower abandonments, 2-group *t* test: *t*_40_ = −2.3, *N* = 76, *p* = 0.027) but there was no difference in the mean number of flower abandonments between flowers with visual components or flowers without visual components (with visual: 5.25 ± 2.79 flower abandonments, *N* = 64, without visual: 5.45 ± 4.11 flower abandonments, 2-group *t* test: *t*_66_ = −0.28, *N* = 42, *p* = 0.781).

### Preference tests

Bees spent less time searching for nectar on artificial flowers with radiating visual patterns over flowers with non-radiating visual patterns (radiating 2.18 ± 0.78 s (mean ± SD), non-radiating 2.67 ± 1.00 s, paired sample *t* test: *t*_*14*_ = 2.44, *p* = 0.029) but there was no difference in nectar discovery time between radiating and non-radiating flowers which had both visual and scent stimuli (radiating 2.6 ± 0.64 s, non-radiating 3.03 ± 1.81 s; paired sample *t* test *t*_14_ = 0.98, *p* = 0.339). There was also no difference in the number of flower visits which did not lead to drinks between flowers with radiating and non-radiating visual patterns (radiating 1.67 ± 1.59 flower visits, non-radiating 2 ± 1.51 flower visits; paired sample *t* test *t*_*14*_ = 0.98, *p* = 0.344), as well as no difference when flowers had both visual and scent stimuli (radiating 3.87 ± 2.7 flower visits, non-radiating 2.67 ± 2.06 flower visits; paired sample *t* test *t*_*14*_ = 1.33, *p* = 0.205). Bumblebees also showed no preference for either radiating or non-radiating visual patterned flowers (one-sample *t* test *t*_*14*_ = 1.5, *p* = 0.155). No preference was found between bimodal radiating or bimodal non-radiating flowers using the same criteria (one sample *t* test *t*_*14*_ = 0.16, *p* = 0.876).

## Discussion

Nectar guides are thought to benefit both plants and their pollinators through reductions in nectar discovery time (NDT) and the likelihood of nectar robbing as well as increasing accuracy and pollen export (Waser and Price 1983; Leonard et al. 2011a, 2013; Hansen et al. 2012). Within this study, we examined the NDTs of forager bumblebees on artificial flowers with different combinations of radiating or non-radiating floral patterns of two modalities: visual and olfactory. We show that the addition of unimodal floral pattern components, whether visual or olfactory, reduces NDT compared to flowers without guides but there was no extra NDT benefit from bimodal displays or radiating patterns during the no-choice speed test. Despite this, NDT was shorter on unimodal flowers with radiating visual patterns than non-radiating visual pattern flowers during preference tests. Our results also suggest that spatial fragrance patterns, both radiating and non-radiating, can be used as nectar guides to reduce nectar discovery times without the aid of visual signals.

During the no-choice speed test, bimodal nectar guides did not have shorter NDTs than unimodal displays, nor were there any time benefits to bees when visiting radiating patterns over non-radiating patterns. These findings suggest that the benefits of bimodal guides may not relate to NDTs. Previous studies have shown that displays incorporating compound signals reduce uncertainty (Leonard et al. 2011a; Riffell and Alarcón 2013) and enhance decision making (Kulahci et al. 2008), although these benefits from multimodal displays relate to pre-landing choices compared to the post-landing nectar discovery speeds investigated in this study. Our findings also suggest that the time benefits of non-radiating nectar guides are comparable, at least with the patterns used here, to radiating guides. This suggest that the benefit of radiating guides, bimodal or unimodal, may relate to other aspects of pollination other than NDT, such as the physical alignment of pollinators on the flower surface which facilitates optimal pollen deposition and placement, conferring fitness benefits to the plant (Scora 1964; Goyret 2010; Hansen et al. 2012). Multimodal signals may also relate to the learning and recall of floral stimuli (Leonard et al. 2011b), the eliciting of feeding behaviours (Raguso and Willis 2002), the facilitation of attention (Talsma et al. 2010), which are not explored in this study. However, these similarities in NDT between the two pattern types may relate to the low level of contrast between the two greens used for visual guide and background. If this is the case, it is possible that patterns with higher degrees of contrast may result in greater differences between pattern types as the chromatic contrast of a guide relates to its effectiveness (Lunau et al. 1996; Dinkel and Lunau 2001). However, bees exposed to purely visual patterns did have shorter NDTs than the control, suggesting the visual guides had sufficient saliency to contribute to a behavioural change.

Our findings also suggest that the ‘increased detection and discrimination hypothesis’ (Hebets and Papaj 2005) does not apply in this scenario and that the addition of any individual display component, whether visual or olfactory, radiating or non-radiating, reduces NDT. This suggests that any visual or olfactory information present on a flower allows for the faster location of rewards by foragers. With this comes the implication that spatial fragrance patterns can be used to reduce nectar discovery times without the aid of visual stimuli, as both unimodal scented flowers had shorter NDTs than the scentless and colourless control. This use of scent patterns as nectar guides has previously been suggested (Bolwig 1954; Bergström et al. 1995; Raguso and Pichersky 1999; Flamini et al. 2002), however reductions in nectar discovery time as suggested in this study have not been shown through experimental methods until now.

Besides the ‘increased detection and discrimination hypothesis’, there are other functional hypotheses relating to multimodal signals which relate to our findings. Reduction in NDT through the addition of either visual or olfactory stimuli also suggests that in environments where display components of certain modalities are compromised there may still be sufficient information on the flower to shorten NDTs. This would support the ‘efficacy backup hypothesis’ which states that individual signals act as a backup to others in varying environmental conditions (Hebets and Papaj 2005). It would also support the ‘redundant signal hypothesis’ which suggests that different signals provide the same information, allowing for increased accuracy of receiver response (Hebets and Papaj 2005).

With our results, we see an example of the bimodal interactions increasing NDT over unimodal displays which could detrimentally affect nectar collection and pollen transfer rates (Table 1). These findings imply that there are interactions between simultaneously presented display components where the olfactory component is negatively affecting the reception or processing of visual component or vice versa. Differences in the colour of flowers have been shown to modulate the learning of scents in hawkmoths (Balkenius and Kelber 2006). If a similar interaction is happening with the bumblebees, it is possible that the colours used affected the reception or processing of the scent or vice versa. However, if this were the case one would expect all combinations of interactions to increase NDT, which is not the case.

**Fig. 3:**
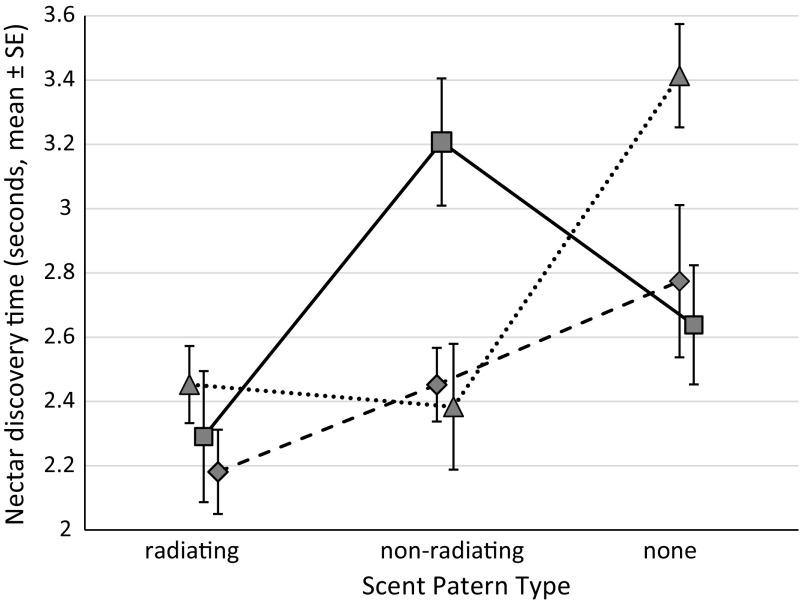
Interaction plot displaying the nectar discover times (mean ± *SE*) of forager bees presented with artificial flowers of varying display elements. *Squares* represent visually radiating patterns, *diamonds* represent visually non-radiating patterns and *triangles* represent patterns without a visual component. Floral displays were comprised of one of three scent arrangements (radiating, non-radiating or no scent) and one of three visual arrangements (radiating, non-radiating or no visual), making nine combinations in total

The spatial arrangement of the bimodal display components also influenced the NDTs. Foragers presented with spatially matched combinations of visual and olfactory stimuli and those presented with combined radiating scent and non-radiating visual patterns have discovery times similar to foragers presented with unimodal patterns. However, foragers presented with non-radiating scent and radiating visual patterns (i.e. spatially unmatched) had longer NDTs than other groups (Fig. 3). This suggests that the spatial arrangement of bimodal signals is an important factor in terms of NDTs. It is worth noting that although non-radiating scent and radiating visual patterns had longer NDTs than other groups, this longer NDT is still shorter than the NDT of the scentless and colourless control, suggesting that a spatially unmatched display is better than no display.

This extended NDT on spatially unmatched flowers is perhaps unsurprising considering that particular volatiles and pigments share biosynthetic pathways and are presented in the same locations on the flower (Zuker et al. 2002; Majetic et al. 2007). However, this relationship between volatile and pigment production, which is apparent in some species, is lacking in others (Olesen and Knudsen 1994; Dormont et al. 2010). With this in mind, it is unclear why one spatially unmatched flower group causes foragers to increase NDTs but not the other, as foragers presented with combined radiating scent and non-radiating visual patterns did not exhibit increased NDTs. This increase in NDT in foragers presented with non-radiating scent and radiating visual patterns highlights the detrimental effects that some display combinations can have on foraging efficiency and highlights the fact that there is still much to be learned about multimodal displays.

During preference tests, no preference for either radiating or non-radiating flower types was found. Previous studies have found radiating elements to be more attractive to bees than plain or circular elements (Free 1970; Lehrer et al. 1995; Shang et al. 2011), while others have shown no preference for artificial flowers with radiating patterns over plain flowers in bee-flies *Usia bicolor* (Johnson and Dafni 1998). It is possible that the lack of preference observed in this study is related to the fact that the greens used for the background and guide were intentionally similar in order to limit bees positioning themselves close to the nectaries when landing (Lunau et al. 2006). This potential limitation in the amount of pre-landing information available to the bee makes it difficult to understand which traits are beneficial to the plant in terms of pollinator preferences.

Despite this lack of preference, results from the preference tests showed that NDTs were shorter on unimodal visually radiating flowers than simultaneously presented non-radiating. This shortened NDT on radiating flowers in preference tests is similar to the results of previous studies where flower to flower flight times and pollinator search times were lower on flowers with guides than plain flowers (Waser and Price 1985; Leonard and Papaj 2011). Our results are also consistent with Johnson and Dafni (1998), in which bombyliid flies (*Usia bicolor*) landing on model flowers with radiating lines walked directly towards the point where the lines converged. The reduced NDTs on artificial flowers comprised of radiating lines which lead to the nectary over ‘disjunct’ guides where radiating lines lead away from the nectary in Goodale et al. (2014) is also reminiscent of our findings. This shortened NDT on radiating flowers in the preference tests suggest there may be time benefits from radiating floral patterns when foragers encounter multiple flower species. If this is the case, pollinators which exhibit low flower constancy may gain a greater advantage from radiating flower patterns, however, more experimental work would need to be done to determine if this is the case.

Although this reduced NDT in radiating patterns was observed in unimodal visual preference tests, the same was not seen between bimodal radiating and non-radiating flowers. This may be due to both flower types having in excess of a minimum information requirement for reward location, though this would need further study to verify. Flowers with non-radiating patterns could still be used to locate rewards as they are still zygomorphic (bilaterally symmetrical), which has been shown to reduce discovery times when compared to asymmetrical flowers (West and Laverty 1998). They may also be well matched to the perceptual systems of bumblebees which primarily visit zygomorphic flower forms (Leppik 1953).

Bees were also found to abandon more unscented flowers than scented flowers. It is possible that the scent acts to provide a foraging context as scent may prompt a foraging response and reduce confusion between different tasks such as locating the nest or within-nest tasks (Leonard and Papaj 2011). This behaviour is similar to tobacco hornworm moths *Manduca sexta,* which do not feed from artificial flowers which lack plant odours (Raguso and Willis 2002). Although bumblebees are known to visit and feed from unscented artificial flowers without difficulty (Keasar et al. 1997), considering odour enhances colour discrimination, memory formation and memory retrieval in bumblebees (Kunze and Gumbert 2001), it is unsurprising that we observe these differences between scented and unscented flowers.

## Conclusion

Within this study, we examined the NDTs of forager bumblebees on artificial flowers with visual, olfactory or combination displays in radiating, non-radiating or mixed arrangements. Our findings suggest that the addition of unimodal floral pattern, whether visual or olfactory or in radiating or non-radiating arrangements, reduces NDT. Bimodal displays did not reduce NDTs compared to unimodal displays and even increased NDTs in one case where the two modalities were spatially unmatched. Radiating patterns also appeared to have no benefit to bees over non-radiating patterns in terms of NDT apart from when both were presented simultaneously during preference tests. These findings imply unimodal signals are sufficient to communicate reward location and that the benefits of bimodal signals are unrelated to NDTs in *B. terrestris*.

## Electronic supplementary material

Below is the link to the electronic supplementary material.

Supplementary material 1 (XLSX 17 kb)

## Abbreviation

NDT: Nectar discovery time
